# Raucherassoziierte interstitielle Lungenerkrankungen

**DOI:** 10.1007/s00117-022-01025-3

**Published:** 2022-06-23

**Authors:** Sebastian Röhrich, Benedikt H. Heidinger, Florian Prayer, Daria Kifjak, Lucian Beer, Christian Wassipaul, Martin Watzenböck, Ruxandra-Iulia Milos, Helmut Prosch

**Affiliations:** 1 Universitätsklinik für Radiologie und Nuklearmedizin, Medizinische Universität Wien, Währinger Gürtel 18–20, 1090 Wien, Österreich; 2 Department of Radiology, UMass Memorial Medical Center and University of Massachusetts Chan Medical School, Worcester, MA USA

**Keywords:** Respiratorische Bronchiolitis, Desquamative interstitielle Pneumonie, Langerhans-Zell-Histiozytose, Idiopathische pulmonale Fibrose, Diagnostische Bildgebung, Respiratory bronchiolitis, Desquamative interstitial pneumonia, Langerhans cell histiocytosis, Idiopathic pulmonary fibrosis, Diagnostic imaging

## Abstract

**Klinisches Problem:**

Raucherassoziierte interstitielle Lungenerkrankungen umfassen heterogene pulmonale Pathologien, deren korrekte Diagnostik prognostische und therapeutische Konsequenzen hat. In diesem Artikel werden die gängigsten raucherassoziierten interstitiellen Lungenerkrankungen beschrieben sowie eine strukturierte Herangehensweise präsentiert, welche den diagnostischen Arbeitsprozess erleichtern kann.

**Empfehlungen für die Praxis:**

Die Computertomographie (CT) besitzt einen hohen Stellenwert in der Diagnose der raucherassoziierten interstitiellen Lungenerkrankungen und kann dazu beitragen, Lungenbiopsien zu verhindern. Um eine hohe diagnostische Genauigkeit zu erreichen, sollten standardisierte Untersuchungsprotokolle sowie eine strukturierte Herangehensweise in der Befundung zur Anwendung kommen. In den entzündlich dominierten Stadien der respiratorischen Bronchiolitis (RB), der respiratorischen Bronchiolitis mit interstitieller Lungenerkrankung (RB-ILD) sowie der desquamativen interstitiellen Pneumonie (DIP) haben die Beendigung des Rauchens sowie Steroide den größten therapeutischen Effekt. Bei fibrotischen Veränderungen (z. B. im Rahmen einer idiopathischen pulmonalen Fibrose [IPF]) können antifibrotische Therapien mit Pirfenidon und Nintedanib zum Einsatz kommen. Patienten mit dem Verdacht auf raucherassoziierte interstitielle Lungenerkrankung sollten in multidisziplinären Boards abgeklärt und behandelt werden.

## Abstract (en)

**Clinical issue:**

Smoking-related interstitial lung diseases are a heterogeneous group of pulmonary abnormalities. The correct diagnosis has prognostic and therapeutic implications. This article introduces the most common smoking-related interstitial lung diseases and describes a structured approach to support the diagnostic workflow.

**Practical recommendations:**

Computed tomography is pivotal in the diagnostic workflow of smoking-related interstitial lung diseases and may reduce the number of unnecessary lung biopsies. To achieve high diagnostic accuracy, a standardized scanning protocol, and a structured assessment approach should be utilized. During inflammatory stages of respiratory bronchiolitis (RB), respiratory bronchiolitis interstitial lung diseases (RB-ILD), and desquamative interstitial pneumonia (DIP), cessation of smoking as well as the use of steroids are the treatment of choice. In case of fibrotic changes (e.g., in idiopathic pulmonary fibrosis [IPF]), antifibrotic therapy with nintedanib and pirfenidone may be used. Patients with suspected smoking-related interstitial lung disease should be discussed in interdisciplinary board meetings.

Die Bildmorphologie der raucherassoziierten interstitiellen Lungenerkrankungen (ILD) stellt eine diagnostische Herausforderung dar, deren konklusive Diagnose eine interdisziplinäre Besprechung erfordert. Eine strukturierte Herangehensweise und adäquate Untersuchungsprotokolle erleichtern hierbei bereits im Vorfeld den anschließenden diagnostischen Prozess (Tab. 1).

**Table 1:** 

Akquisitionstechnik	
Schichtdicke	1 mm (zumindest < 1,5 mm)
Matrix	Mindestens 768 × 768
Rekonstruktions-Kernel	Hohe Ortsauflösung (Lunge oder Knochen)
Röhrenspannung	120 kV
Röhrenstrom	(100–200 mAs), oft bereits automatisch moduliert
Positionierung	Rücken- und Bauchlage
Inspirationstiefe	Tiefe Inspiration und Exspiration

## Hintergrund und Definition

Während die häufigsten raucherassoziierten Lungenerkrankungen die chronisch-obstruktive Lungenerkrankung (COPD) und das Lungenkarzinom sind, gibt es eine Gruppe an Erkrankungen, welche primäre Veränderungen des interstitiellen Lungengewebes darstellt (Tab. 2). Hierzu zählen die respiratorische Bronchiolitis (RB), die respiratorische Bronchiolitis mit interstitieller Lungenerkrankung (RB-ILD) und die desquamative interstitielle Pneumonie (DIP). Diese vermeintlich separaten Begriffe entsprechen eher einem Spektrum ein und derselben Erkrankung in verschiedenen Stadien. Zu weiteren interstitiellen Lungenerkrankungen, für welche Rauchen einen wichtigen Risikofaktor darstellt, gehören die pulmonale Langerhans-Zell-Histiozytose, die kombinierte Lungenfibrose mit Emphysem (CPFE), die idiopathische Lungenfibrose (IPF), die raucherassoziierte interstitielle Fibrose (SRIF) sowie die akute eosinophile Pneumonie. Andere Erkrankungen, bei welchen eine Beteiligung des pulmonalen Interstitiums eine Rolle spielt und für welche ebenso Rauchen als Risikofaktor identifiziert wurde, sind diffuse alveoläre Hämorrhagien bei Goodpasture-Syndrom und die pulmonale alveoläre Proteinose.

**Table 2:** 

Erkrankung	RB	RB-ILD	DIP	LCH
Epidemiologie	98 % aller Raucher*innen weisen eine RB auf	30–40 Jahre	30–60 Jahre	20–40 Jahre
	–	> 30 PY	> 18 PY	95 % Rauchen in der Anamnese
	–	–	M:W = 2:1	M:W = 2:1
Prädominantes Muster	Schlecht abgrenzbare, zentrilobuläre Milchglasnoduli	Zentrilobuläre Milchglasnoduli Fleckiges bis diffuses Milchglas	Diffuses Milchglas	Zentrilobuläre, peribronchovaskuläre Noduli (evtl. mit Kavernen) Bizarr konfigurierte Zysten
Verteilung des prädominanten Musters	Oberlappenbetont	Ober- und Unterlappen sind gleich betroffen	Unterlappenbetont	Oberlappenbetont
Zusätzliche Befunde in der Bildgebung	Bronchialwandverdickungen	Bronchialwandverdickungen	Bronchialwandverdickungen	Verdickte interlobuläre Septen
	Emphysem	Emphysem	Emphysem	Emphysem
	–	Periphere Fibrose (selten; unterlappenbetont)	Periphere Fibrose (bis zu 50 %; unterlappenbetont)	Fibrose (Endstadium)
	–	Fleckige Areale erhöhter Transparenz (unterlappenbetont)	–	–
Pathologisches Korrelat	Chronische Inflammation und pigmentierte Makrophagen in Bronchiolarwänden	Peribronchiale Inflammation und pigmentierte Makrophagen in Alveolen	Ausgeprägte Ansammlungen von pigmentierten Makrophagen in den Alveolen	Granulome mit umgebender Fibrose, welche kavernieren und eine Evolution zu dünnwandigen Zysten durchlaufen
	Evtl. geringe Fibrose der Bronchiolarwände	Fibrose der Alveolen	–	Evtl. myeloide neoplastische Zellen mit Inflammation
Symptome	Oft asymptomatisch	Chronischer Husten	Chronischer Husten	Chronischer Husten
	–	Kurzatmigkeit	Kurzatmigkeit	Kurzatmigkeit
	–	–	–	Spontaner Pneumothorax
Lungenfunktion	Oft normal	Keine oder nur geringe Restriktion	Keine oder nur geringe Restriktion	Obstruktion, Restriktion oder gemischt
	–	DLCO reduziert	DLCO reduziert	DLCO reduziert
Differenzialdiagnosen	EAA Follikuläre Bronchiolitis Atypisches Bild einer Sarkoidose Vaskulitis	NSIP EAA	NSIP	*Früh (Noduli)* GPA Sarkoidose Metastasen Miliare Tbc
	–	–	–	*Später (Zysten)* LAM Emphysem LIP

*RB* respiratorische Bronchiolitis, *RB-ILD* respiratorische Bronchiolitis mit interstitieller Lungenerkrankung, *DIP* desquamative interstitielle Pneumonie, *LCH* Langerhans-Zell-Histiozytose, *M* männlich, *W* weiblich, *PY* Pack Years, *DLCO* Kohlenmonoxid-Diffusionskapazität, *GPA* granulomatöse Polyangiitis, *Tbc* Tuberkulose, *LAM* Lymphangioleiomyomatose, *LIP* lymphoide interstitielle Pneumonie, *EAA* Exogen allergische Alveolitis, *NSIP* nichtspezifische interstitielle Pneumonie

## Respiratorische Bronchiolitis (mit interstitieller Lungenerkrankung) und desquamative interstitielle Pneumonie

Wie bereits erwähnt, entsprechen die RB, RB-ILD und DIP einem Erkrankungsspektrum. Bei der RB handelt es sich um eine chronische Entzündung der Bronchiolen und angrenzenden Alveolen, welche mit einer vermehrten Ansammlung an pigmentierten Makrophagen einhergeht [6, 10]. Bei aktiven Raucher*innen findet sich unabhängig vom Vorliegen potenzieller Symptome oder pathologischer bildgebender Befunde das histologische Bild einer RB im Sinne eines Reaktionsmusters der Lunge auf diese Noxe [11]. Histologisch können sich auch kleine Areale fibrotischer Veränderungen finden, welche jedoch keine fibrosetypischen Symptome verursachen und auch nicht als solche in der Bildgebung zu erkennen sind [6]. Die RB wird als eine Frühform der RB-ILD angesehen und befindet sich damit innerhalb eines gemeinsamen Spektrums mit der DIP. Während die RB jedoch fast ausschließlich bei Raucher*innen vorkommt, gibt es bei der DIP auch seltene andere Ätiologien [12]. Die RB ist oft asymptomatisch und entspricht in der CT dann einem Zufallsbefund.

**Figure 1:**
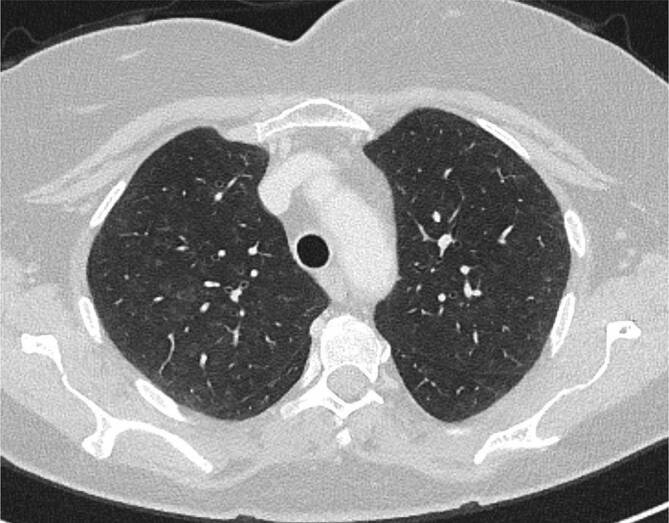

Bildmorphologisch finden sich bei der RB typischerweise schlecht abgrenzbare, zentrilobuläre Milchglasnoduli mit einer oberlappenbetonten Verteilung ([6]; Abb. 1). Differenzialdiagnostisch sollte bei chronisch bestehenden, diffusen, zentrilobulären Milchglasnoduli noch an die exogen allergische Alveolitis (EAA), eine follikuläre Bronchiolitis oder an das atypische Bild einer pulmonalen Sarkoidose gedacht werden. Sollte die Chronizität der Veränderungen unklar sein, kommen an akuten Differenzialdiagnosen noch Infekte sowie Vaskulitiden hinzu.

**Figure 2:**
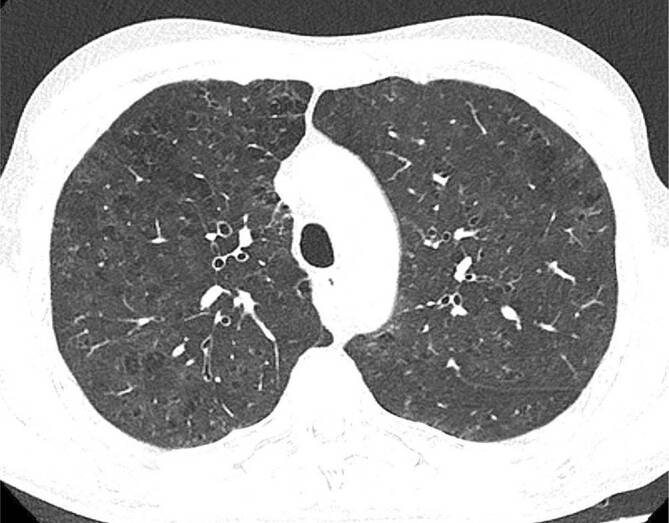

Bei Fortschreiten der Erkrankung mit stärker ausgeprägten Symptomen wie Kurzatmigkeit, Dyspnoe und chronischem Husten wird von der RB-ILD gesprochen. Eventuell sind radiologische und histologische Veränderungen deutlicher erkennbar, jedoch ist eine Unterscheidung zwischen RB und RB-ILD in der CT nicht möglich. Fibrotische Veränderungen sind histologisch hier auch abseits der direkt betroffenen Bronchiolen bis in die Alveolen zu finden. Diese umfassenderen Veränderungen erklären das CT-Erscheinungsbild der RB-ILD, welche als prädominantes Muster flächige, konfluierende Milchglasareale aufweist (Abb. 2). Die in der RB vorherrschenden, zentrilobulären Milchglasnoduli sind nur noch als sekundärer Bildbefund in weniger stark betroffenen Lungenarealen zu verstehen. Die Verteilung ist nicht mehr klar oberlappenbetont wie bei der RB, sondern umfasst teils auch weiter kaudal gelegene Abschnitte, was die Annäherung an die unterlappenbetonte DIP im Spektrum der raucherassoziierten interstitiellen Lungenerkrankungen verdeutlicht [13, 14]. Fortgeschrittene fibrotische Veränderungen, falls vorhanden, finden sich peripher in den subpleuralen Abschnitten der Lunge. Als weitere sekundäre Bildmerkmale finden sich typische raucherassoziierte Veränderungen wie Bronchialwandverdickungen und hypodense Areale in den basalen Lungen im Sinne einer Erkrankung der kleinen Atemwege mit Air-Trapping [15] sowie zentrilobuläres Lungenemphysem.

Die RB-ILD wird zunehmend ohne Lungenbiopsie diagnostiziert, wenn die typischen CT-Veränderungen sowie pigmentierte Makrophagen in der bronchoalveolären Lavage vorliegen und keine Lymphozytose besteht. Diese würde eher für eine EAA sprechen, die bei Raucher*innen sehr selten ist [16, 17].

Sowohl für die RB als auch die RB-ILD besteht die Therapie der Wahl in der Beendigung des Rauchens, jedoch wurde für einen kleinen Anteil an Personen eine Progression der Erkrankung trotz Beendigung des Rauchens beschrieben [12]. Eine Verlaufskontrolle nach 3 bis 6 Monaten sollte zur Bestätigung der Regredienz oder vollständigen Rückbildung der Lungenveränderungen durchgeführt werden [12].

**Figure 3:**
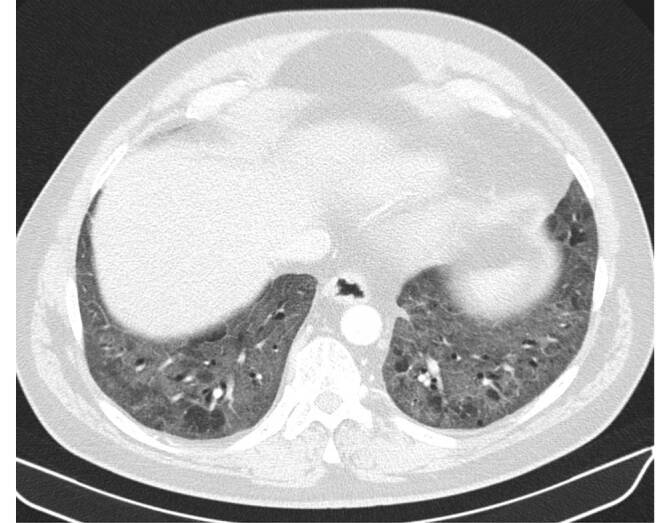

Die desquamative interstitielle Pneumonie (DIP) weist letztlich zu 90 % eine Nikotinanamnese auf; es existieren jedoch auch andere Risikofaktoren (z. B. autoimmune und rheumatologische Erkrankungen, humanes Immundefizienzvirus (HIV), eine Medikamentenassoziation sowie Umweltfaktoren wie Asbestexposition; [2]). Histopathologisch finden sich wie bei der RB und RB-ILD pigmentierte Makrophagen. Das prädominante CT-morphologische Muster der DIP ist diffuses Milchglas (Abb. 3). Dieses tritt in den meisten Fällen bilateral, symmetrisch und unterlappenbetont auf. Seltener ist auch eine fleckige Verteilung möglich [2]. Zusätzliche Bildmerkmale sind kleine hypodense Aussparungen im Milchglas bzw. Emphysemareale und Bronchialwandverdickungen sowie irreguläre Retikulierungen und kleinzystische Veränderungen im Rahmen einer etwaigen Fibrosierung. Die radiologische Abgrenzung zu einer RB-ILD kann schwer oder gänzlich unmöglich sein. Therapeutisch steht die Beendigung des Rauchens im Vordergrund, welche mit einer guten Prognose einhergeht.

## Langerhans-Zell-Histiozytose

Die Langerhans-Zell-Histiozytose ist eine myeloische Neoplasie mit einer inflammatorischen Komponente, die nahezu alle Organe betreffen kann. Bei Erwachsenen betrifft die pulmonale LCH vorwiegend junge, 20- bis 40-jährige Raucher*innen [3, 6]. In den meisten Fällen sind diese Patient*innen asymptomatisch oder präsentieren sich mit unspezifischen Symptomen wie Dyspnoe und nichtproduktivem Husten. Manchmal ist ein spontaner Pneumothorax das initiale Symptom, weshalb hier bei passender Anamnese an eine mögliche, zugrundeliegende LCH gedacht werden sollte.

**Figure 4:**
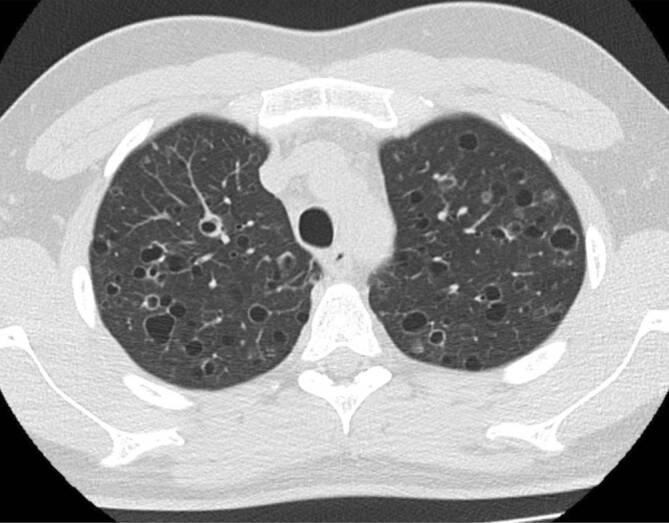

**Figure 5:**
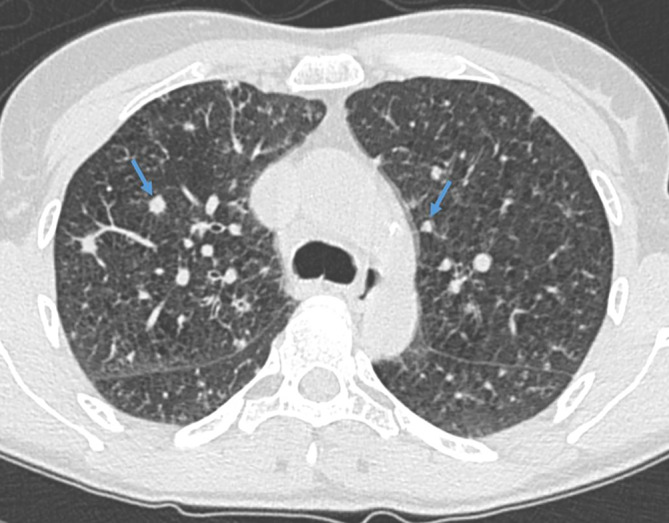

Histopathologisch findet man die namensgebenden Langerhans-Zellen, welche im Epithel der Bronchiolen proliferieren, zu einer direkt am Rand der Bronchiolen angrenzenden Fibrose führen und schließlich als klein-noduläre Veränderungen in der CT zu sehen sind ([4]; Abb. 4). Von den fibrotischen Veränderungen nimmt man an, dass sie über eine narbige Verziehung an den Bronchiolen zu den bizarr geformten Zysten führen, welche gemeinsam mit den Noduli das charakteristische Bild der LCH darstellen (Abb. 5). Als mögliche Genese werden in der Literatur sowohl immunmediierte [3] als auch myeloisch-neoplastische Prozesse diskutiert [5].

Im zeitlichen Verlauf der pulmonalen LCH können bildgebend unterschiedliche Merkmale überwiegen und auch koexistieren, beginnend bei den anfänglichen Noduli, bis hin zu dünnwandigen Zysten. Die zentrilobulär gelegenen Noduli haben einen Durchmesser von ca. 1–5 mm und weisen aufgrund der direkt umgebenden fibrotischen Reaktion eine irreguläre Berandung auf. Sie sind von regulärem Lungengewebe umgeben, da dieses im Gegensatz zu anderen interstitiellen Lungenerkrankungen nicht pathologisch verändert ist [3]. Erschwert wird die Diagnose durch die unterschiedliche Ausprägung der Noduli, welche von nur einzelnen wenigen, bis hin zu unzähligen reichen können. Die später hinzukommenden Zysten beginnen als kleine Kavernen mit dicken Wänden, welche größer (bis zu 2 cm, normalerweise aber < 1 cm) und damit dünnwandiger werden [3]. Ein Konfluieren von mehreren Zysten verstärkt neben der irregulären Berandung den *bizarren* Eindruck. Die LCH ist oberlappenbetont mit Aussparung der kostophrenischen Winkel, was differenzialdiagnostisch wertvoll ist [3, 4, 6].

Andere, nicht prädominante bildgebende Merkmale sind Milchglasveränderungen, verdickte interlobuläre Septen, Emphysem (wie bei allen raucherassoziierten Lungenerkrankungen) sowie ein Mosaikmuster [4, 6]. Bei sehr weit fortgeschrittenen Erkrankungen können Zeichen der Lungenfibrose mit *Honeycombing* überwiegen, welche in diesen Stadien dann nur schwer von anderen Formen der Lungenfibrose zu unterscheiden sind.

Die Differenzialdiagnosen entsprechen dem vorwiegenden Muster des jeweiligen Stadiums. Zu Beginn überwiegen Noduli, weshalb die LCH hier von granulomatösen Erkrankungen wie der granulomatösen Polyangiitis (GPA) sowie der Sarkoidose und der miliaren Tuberkulose unterschieden werden muss. Des Weiteren sollte eine onkologische Grunderkrankung an Metastasen denken lassen.

Sobald multiple Zysten das prädominante Muster darstellen, verändert sich die Liste der Differenzialdiagnosen entsprechend. Die Lymphangioleiomyomatose (LAM) weist uniforme, rundliche Zysten auf, welche im Gegensatz zur LCH ohne bestimmtes Verteilungsmuster auftreten und somit auch die Unterlappen betreffen. Die Pneumocystis-jirovecii-Pneumonie (PCP) kann mit Zysten einhergehen, das prädominante Muster sind hier jedoch Milchglasveränderungen [18]. Zystische Bronchiektasien, wie sie bei der allergischen bronchopulmonalen Aspergillose (ABPA) auftreten, sind zentral gelegen, gut den großen Bronchien zuzuordnen und weisen sehr dichten Mukus auf [19]. Die lymphozytische interstitielle Pneumonie (LIP) kann als Erscheinungsform ausschließlich Zysten aufweisen, welche dann glatt berandet sowie dünnwandig sind. Sie kommt bei Autoimmunerkrankungen sowie Immunsuppression vor [20].

Die wichtigste therapeutische Maßnahme ist das Beenden des Rauchens. Ansonsten können Kortikosteroide zu einer Besserung führen. In schweren und progredienten Fällen steht als Ultima Ratio die Lungentransplantation zur Verfügung [3, 4].

## Kombinierte pulmonale Fibrose und Emphysem

Die kombinierte pulmonale Fibrose mit Emphysem (CPFE) wird in der Literatur als mögliche Ausprägung der raucherassoziierten interstitiellen Lungenerkrankungen diskutiert [21]. Bereits für die idiopathische pulmonale Fibrose (IPF) wurde ein Zusammenhang mit dem Rauchen beschrieben [22]. Bei der CPFE ist der zugrundeliegende Pathomechanismus noch nicht geklärt, es scheint aber Menschen zu geben, welche ein erhöhtes Risiko zur Entwicklung einer Lungenfibrose durch das Rauchen aufweisen.

Die Relevanz der CPFE besteht in dem erhöhten Risiko von Komorbiditäten im Vergleich zur alleinigen Ausprägung der Fibrose oder des Emphysems. Aufgrund der diffusen Zerstörung des Lungenparenchyms ist bei der CPFE mit einer noch höheren Prävalenz der pulmonalen Hypertension zu rechnen als bei dem alleinigen Vorliegen einer IPF [21]. Darüber hinaus besteht ein erhöhtes Risiko, Plattenepithelkarzinome der Lunge mit einer besonders schlechten Prognose zu entwickeln [23].

**Figure 6:**
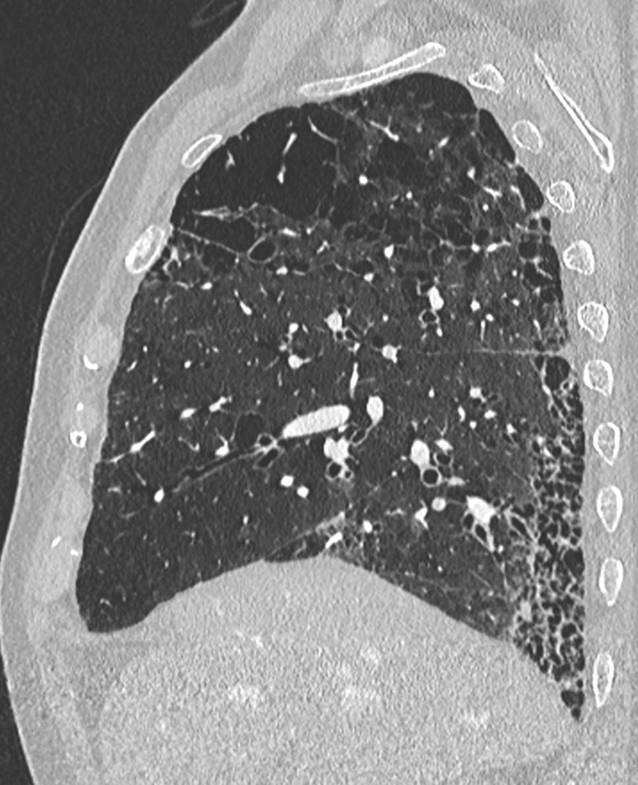

In der Bildgebung lassen sich die beiden namensgebenden Muster beobachten (Abb. 6). Oberlappenbetont finden sich ausgedehnte Areale des vor allem paraseptalen Lungenemphysems (geringer auch des zentrilobulären Emphysems). Unterlappenbetont finden sich die fibrotischen Areale, entweder als Muster einer „usual interstitial pneumonia“ (UIP) oder „nonspecific interstitial pneumonia“ (NSIP). Diagnostische Schwierigkeiten ergeben sich beim Erkennen eines eventuell vorhandenen und prognostisch relevanten *Honeycombings*, welches nach kranial hin mit dem Emphysem konfluiert und dann schwierig von diesem zu differenzieren sein kann [24, 25].

Letztlich ist die CPFE noch unspezifisch definiert und umfasst verschiedene fibrotische Entitäten. So kann angenommen werden, dass es eine Subgruppe der CPFE gibt, welche ein Emphysem mit koexistierender IPF aufweist, im Gegensatz zu denen, welche eine raucherassoziierte interstitielle Fibrose („smoking-related interstitial fibrosis“, SRIF) aufweisen. Dies wäre eine Erklärung für die variable Prognose der CPFE [26, 27]. In den folgenden Abschnitten werden die IPF und SRIF weiter diskutiert.

## Raucherassoziierte interstitielle Fibrose

**Figure 7:**
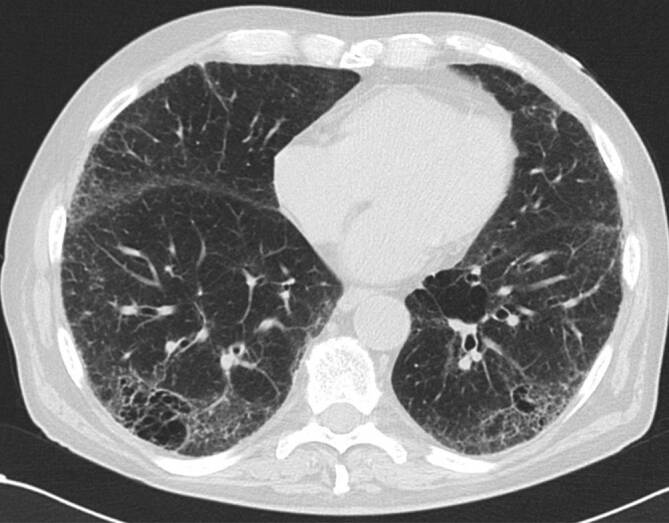

Die SRIF ist ein häufiger histologischer Befund in Lungenpräparaten von (Ex‑)Raucher*innen [28]. Sie muss von prognostisch deutlich schlechteren Diagnosen, wie der IPF, differenziert werden. Pathologisch charakterisiert sich die SRIF durch dicke hyalinisierte alveoläre Septen mit hyperplastischen glatten Muskelbündeln. Diese Fibrose geht mit anderen raucherassoziierten Veränderungen einher, wie dem Emphysem und der respiratorischen Bronchiolitis ([28]; Abb. 7). Typische histologische Veränderungen der UIP, wie das *Honeycombing*, fehlen hierbei.

Nicht immer existieren CT-morphologisch erkennbare Veränderungen in histologisch verifizierten SRIF-Fällen. Wenn diese in Studien mit radiologisch-histologischer Korrelation beschrieben wurden, dann handelte es sich dabei um mikronoduläre Veränderungen sowie Milchglasareale [29], jedoch ist hier eine klare Differenzierung von SRIF zu anderen raucherassoziierten interstitiellen Lungenveränderungen (z. B. der RB) nicht möglich. In Fällen mit deutlicher ausgeprägten fibrotischen Veränderungen und Honeycombing könnte eine Klassifizierung von SRIF gegenüber der UIP gelingen, indem das Verhältnis zwischen Emphysem- und Honeycombing-Ausdehnung bestimmt wird. Hierbei zeigt sich eine größere Rate zugunsten des Emphysems in Fällen der SRIF verglichen mit der UIP [27]. Weitere Bildmerkmale, die mit der SRIF korrelieren sind eine Aussparung der kostophrenischen Winkel durch das Honeycombing, eine asymmetrische Verteilung des Honeycombing sowie ein Fehlen von Honeycombing in den Oberlappen [27, 30].

Wie bereits oben erwähnt, ist die Unterscheidung zwischen SRIF und IPF prognostisch relevant, da die 5‑Jahres-Überlebensrate deutlich variiert (SRIF 85,7 % vs. IPF 40,7 %; [27]).

## Idiopathische pulmonale Fibrose

**Figure 8:**
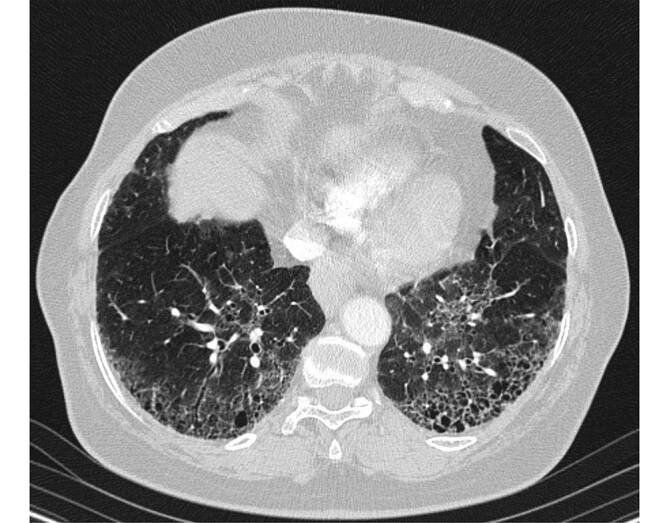

**Figure 9:**
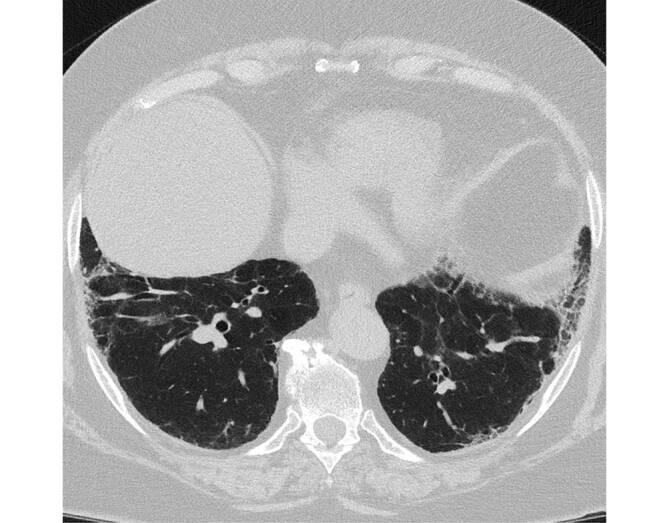

Die IPF ist die häufigste und mit der größten Mortalität behaftete pulmonale Fibrose. Am häufigsten sind Männer über 60 Jahren betroffen, genetisch Risikofaktoren können aber ein jüngeres Erkrankungsalter im Rahmen der familiären IPF bedingen [31]. Rauchen stellt einen Risikofaktor für die Entstehung der IPF dar, welcher in 60 % der IPF-Fälle anamnestisch erhoben werden kann [22]. Die Diagnose erfolgt durch das histologische oder radiologische Muster der UIP sowie das Fehlen anderer Ursachen (z. B. rheumatische Erkrankungen, Medikamententoxizität oder Umweltfaktoren wie Asbestexposition; [32]). Das typische UIP-Muster (Abb. 8) besteht aus einer basalen und subpleuralen Verteilung von Honeycombing mit irregulärer Retikulierung (und ggf. Traktionsbronchiektasien) sowie dem Fehlen von atypischen Mustern (z. B. ausgeprägten Milchglasarealen). Eine Biopsie ist bei Vorliegen eines typischen UIP-Musters nur bei nicht zur IPF passenden Klinik notwendig. Ein wahrscheinliches UIP-Muster (Abb. 9 und 10) setzt sich aus denselben Merkmalen wie das typische UIP-Muster zusammen, jedoch fehlt Honeycombing. Hier besteht bei klinischer Unsicherheit die Notwendigkeit zur Biopsie. Letztlich gibt es Bildmerkmale, welche mit einer IPF nicht konsistent sind; diese umfassen eine Oberlappenbetonung, eine peribronchovaskuläre Verteilung, Konsolidierungen oder Milchglasareale als prädominantes Muster, ausgeprägtes Mosaikmuster, Air-Trapping, Rundherde sowie Zysten. In diesen Fällen sind die Biopsie sowie eine Besprechung in interdisziplinären Boards notwendig [33].

**Figure 10:**
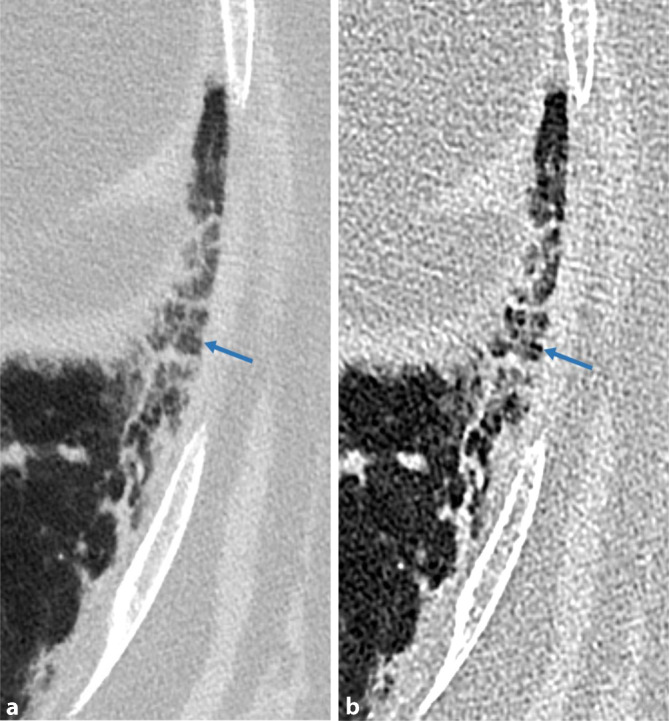

Das mediane Überleben ohne Therapie liegt bei 2,5 bis 3,5 Jahren [34], therapeutisch können antifibrotische Medikamente wie Pirfenidone oder Nintedanib das Voranschreiten der IPF jedoch verlangsamen [35].

## Andere ILD mit Rauchen als Risikofaktor

Mehrere Erkrankungen weisen eine erhöhte Prävalenz bei Raucher*innen auf. Dazu gehören die akute eosinophile Pneumonie (AEP), welche durch Rauchen ausgelöst werden kann [36], die mit der rheumatoiden Arthritis (RA) vergesellschaftete ILD (RA-ILD; [37]), die diffuse alveolare Hämorrhagie im Rahmen des Goodpasture-Syndroms [38] sowie die pulmonale Alveolarproteinose (PAP; [39]).

## Fazit für die Praxis

- Die wichtigsten raucherassoziierten interstitiellen Lungenerkrankungen umfassen das Spektrum der respiratorischen Bronchiolitis (RB), respiratorischen Bronchiolitis mit interstitieller Lungenerkrankung (RB-ILD), desquamativen interstitiellen Pneumonie (DIP), Langerhans-Zell-Histiozytose (LCH) sowie der kombinierten Lungenfibrose mit Emphysem (CPFE), raucherassoziierten interstitiellen Fibrose (SRIF) und idiopathischen pulmonalen Fibrose (IPF).
- Insbesondere die CPFE weist eine sehr heterogene Prognose auf und bietet Gelegenheit zur weiteren Evaluierung, um die Differenzierung der prognostisch ungünstigen IPF von weniger letalen Pathologien wie der SRIF zu unterstützen.
- Generell sollten unklare Fälle im interdisziplinären Rahmen diskutiert und ggf. einer histologischen Abklärung zugeführt werden.
- Therapeutisch steht eine Beendigung des Rauchens klar im Vordergrund.
- Steroide können inflammatorische Prozesse reduzieren.
- Als Ultima Ratio steht die Lungentransplantation zur Verfügung.
